# Individual-based modeling unravels spatial and social interactions in bacterial communities

**DOI:** 10.1093/ismejo/wraf116

**Published:** 2025-08-21

**Authors:** Jian Wang, Ihab Hashem, Satyajeet Bhonsale, Jan F M Van Impe

**Affiliations:** BioTeC+, Chemical and Biochemical Process Technology and Control, Department of Chemical Engineering, Faculty of Engineering Technology, KU Leuven, 9000 Ghent, Belgium; BioTeC+, Chemical and Biochemical Process Technology and Control, Department of Chemical Engineering, Faculty of Engineering Technology, KU Leuven, 9000 Ghent, Belgium; BioTeC+, Chemical and Biochemical Process Technology and Control, Department of Chemical Engineering, Faculty of Engineering Technology, KU Leuven, 9000 Ghent, Belgium; BioTeC+, Chemical and Biochemical Process Technology and Control, Department of Chemical Engineering, Faculty of Engineering Technology, KU Leuven, 9000 Ghent, Belgium

**Keywords:** individual-based modeling (IbM), social interactions, spatial interactions, individual variability, spatial heterogeneity, adaptive strategies, niche differentiation, metabolic cooperation, resource gradient

## Abstract

Bacterial interactions are fundamental in shaping community structure and function, driving processes that range from plastic degradation in marine ecosystems to dynamics within the human gut microbiome. Yet, studying these interactions is challenging due to difficulties in resolving spatiotemporal scales, quantifying interaction strengths, and integrating intrinsic cellular behaviors with extrinsic environmental conditions. Individual-based modeling addresses these challenges through single-cell-level simulations that explicitly model growth, division, motility, and environmental responses. By capturing both the spatial organization and social interactions, individual-based modeling reveals how microbial interactions and environmental gradients collectively shape community architecture, species coexistence, and adaptive responses. In particular, individual-based modeling provides mechanistic insights into how social behaviors—such as competition, metabolic cooperation, and quorum sensing—are regulated by spatial structure, uncovering the interplay between localized interactions and emergent community properties. In this review, we synthesize recent applications of individual-based modeling in studying bacterial spatial and social interactions, highlighting how their interplay governs community stability, diversity, and resilience. By linking individual-scale interactions with the ecosystem-level organization, individual-based modeling offers a predictive framework for understanding microbial ecology and informing strategies for controlling and engineering bacterial consortia in both natural and applied settings.

## Introduction

**Figure 1:**
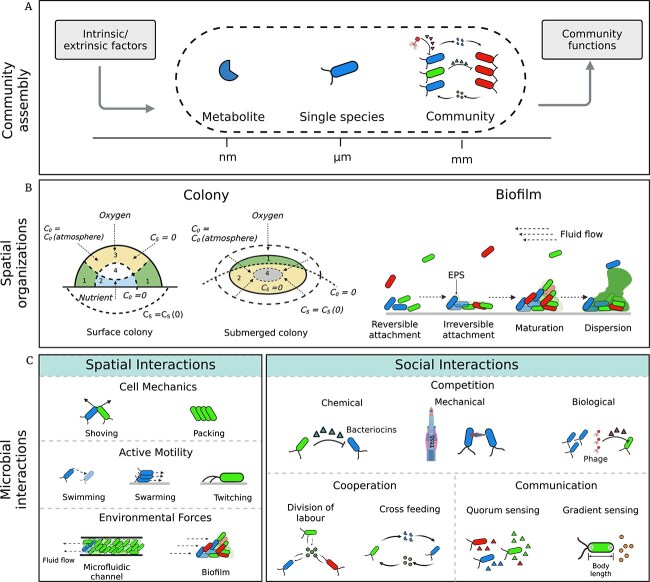
Bacterial organizations emerge from spatial and social interactions. (A) Scales of bacterial interactions. Bacterial interactions occur across molecular (nm), single-species (μm), and community (mm) scales. At the community scale, multispecies interactions and environmental factors drive the emergence of complex processes that shape ecosystem functions. The design of this panel was inspired by Gralka *et al*. [10]. (B) Bacterial organizations. Colonies and biofilms represent two major bacterial organizations. Colonies can grow on surfaces or be submerged in a medium, developing different growth zones based on nutrient and oxygen availability. Zone 1 has sufficient nutrients and oxygen; zone 2 has sufficient nutrients but depleted oxygen; zone 3 has sufficient oxygen but depleted nutrients; zone 4: both nutrients and oxygen are depleted (figure taken from Dens *et al*. [11]). Biofilm forms in four stages: (i) attachment, where cells attach to surfaces; (ii) early formation, where clusters grow through division, cooperation, and competition; (iii) maturation, where biofilms develop into EPS-stabilized structures; (iv) detachment, where cells disperse to colonize new surfaces. (C) Spatial and social interactions. Spatial interactions arise as cells adapt to physical constraints from growth, division, and environmental pressures. Social interactions occur when cells release and sense chemical molecules in their environment.

In nature, bacteria rarely live in isolation. Instead, they form intricate communities where biotic and abiotic factors synergistically drive the emergence of complex spatial patterns (Fig. 1A) [1]. Colonies and biofilms represent two primary forms of bacterial communities. Colonies, which form on surfaces or in liquid media (e.g. submerged colonies), typically begin as monolayers and then develop into three-dimensional (3D) structures as central resources become limited (Fig. 1B). Biofilms, by contrast, exhibit greater spatial heterogeneity and niche differentiation, enabling species with diverse metabolic capabilities to coexist within an extracellular polymeric substance (EPS) matrix (Fig. 1B). Multispecies coexistence within bacterial communities gives rise to diverse inter- and intra-species interactions. These interactions occur through competitive or cooperative behaviors and communication, all of which are shaped by spatial structure and environmental conditions [2]. Competition may arise via (i) diffusible inhibition (i.e. long-range interactions) [3], (ii) contact-dependent inhibition (i.e. short-range interactions) [4], and (iii) exploitation of resources and space [5]. Cooperation, on the other hand, occurs through mechanisms such as (i) colonization and aggregation [6], (ii) metabolic cross-feeding [7], and (iii) induction of phenotypic gene expression in neighboring cells, which improves the resistance of multispecies biofilms to environmental stressors compared to single-species biofilms [8]. These spatially structured interactions create localized ecological pressures and raise fundamental questions about how microbial communities maintain resilience, species diversity, and functional stability [9]. Answering such questions requires deeper insight into bacterial cellular interactions and their collective emergent properties.

Technological advances—such as time-resolved 3D live-cell imaging [12], mass spectrometry imaging [13], multiparameter flow cytometry [14], and single-cell transcriptomics [15]—now allow macroscopic bacterial communities to be spatially resolved at the single-cell level. However, major challenges remain in linking individual-level interactions to emergent community-level properties [16], primarily due to the difficulty in delineating spatial and temporal scales [17], quantifying interaction strength [18], and accounting for the combined effects of extrinsic and intrinsic factors [19]. Individual-based modeling (IbM) offers a powerful approach to address these challenges [20]. By representing each microorganism as a discrete entity with specific traits and behaviors, IbM captures the impact of spatial constraints, local resource availability, and individual variability on emergent population dynamics [21–23]. This bottom-up approach has proven valuable, as bacterial communities are increasingly recognized not as simply the additive sum of homogeneous populations but as complex assemblages where individual-level interactions decisively shape community dynamics [24–26]. Recent applications of IbM have demonstrated its effectiveness in understanding key ecological processes, from predicting how nutrient gradients drive species segregation, niche partitioning, and metabolic stratification in bacterial colonies [27–29], to revealing how cell-to-cell signaling mechanisms influence biofilm architecture, interspecies cooperation, and spatial organization of competitive and mutualistic interactions [30–32], as well as how mechanical forces shape community structure, regulate access to spatial niches, and support biofilm resilience under fluctuating environmental conditions [33–35]. Taken together, these studies demonstrate IbM’s ability to bridge local cellular behavior with emergent community-level organization and have provided critical insights into how spatial constraints regulate competition, cooperation, and communication within bacterial communities.

Given IbM’s growing importance in microbial ecology, several reviews have explored different facets of the approach. Hellweger *et al*. discussed IbM’s functionalities and its significance in integrating datasets across scales [36]. Kreft *et al*. provided an update summarizing IbM applications in resolving microbial intracellular heterogeneity [37]. Nagarajan *et al*. examined state-of-the-art IbM algorithms for simulating intracellular processes, single-cell behaviors, and both cell–cell and cell–environment interactions [38]. Henderson *et al*. reviewed the biological, chemical, and physical factors that affect spatial patterning and proposed modeling frameworks, including IbM, to predict spatial organization in microbial communities [9].

Building upon these important groundworks, we synthesize how IbM reveals the complex interplay between spatial and social interactions in structuring bacterial communities and shaping their ecological dynamics. First, we analyze the roles of spatial and social interactions in shaping bacterial community organization. Second, we review recent applications of IbM in studying bacterial spatial and social interactions. Finally, we discuss the integration of multiscale datasets to improve IbM calibration and predictive power. This review provides a timely synthesis for researchers aiming to understand and predict bacterial community dynamics at the single-cell level, and to foster strategies for controlling and engineering bacterial consortia. Comprehensive reviews are available for readers seeking deeper insights into specific interaction mechanisms, including microbial competition [39–41], cooperation and metabolic cross-feeding [42–44], and communication [45, 46]. Additional guidelines on IbM design and implementation are provided in [47–49].

## Interactions shape bacterial communities

The organization of bacterial communities emerges from two fundamental types of interactions: (i) spatial interactions, driven by mechanical forces between cells and their environment [50]; and (ii) social interactions, mediated by chemical signals and metabolic exchanges [51] (Fig. 1C). Spatial interactions, including cellular mechanics, active motility, and environmental constraints, influence the spatial arrangement of cells and the formation of localized resource gradients. Social interactions, including competition, cooperation, and communication, determine how cells share or compete for these resources. Together, these interactions shape the structure and functionality of bacterial communities and are influenced by several extrinsic and intrinsic factors, such as spatial heterogeneity, individual variability, and adaptive strategies [52, 53]. Recent studies have further shown that interaction strength [54], interaction range [55], and interaction type [16] can significantly affect the development and spatial organization of bacterial communities [17].

### Spatial interactions

Bacterial growth is shaped by three interdependent biophysical drivers: cellular mechanics [56–58], active motility [59–61], and environmental constraints [62–64]. Within dense communities, cell growth and division generate mechanical stresses that propagate through cell-to-cell contact networks [65]. These mechanical stresses induce distinct spatial organization by influencing cell packing, displacement, and segregation. For example, flower-like patterns have been observed in mixed-species colonies, where highly motile species (*Escherichia coli*) physically push outward on non-motile species (*Acinetobacter baylyi*) [66]. Branching patterns emerge in *Bacillus subtilis* colonies under nutrient-limited conditions, where individual cells undergo stress-induced deformation, resulting in dynamic structural rearrangements [67]. Vertically stratified layers form in *Pseudomonas aeruginosa* biofilms, where compressive forces from cellular growth and division lead to variation in cell density and metabolic activity along the vertical axis [68]. Recent studies have shown that such mechanical interactions can propagate across hundreds of micrometers to create long-range order within bacterial communities [69, 70].

Active motility enhances spatial organization by enabling individual cells and collectives to relocate. For example, swimming, driven by flagella, allows rapid movement of individual bacterial cells in liquid media [59]. Swarming, which depends on both flagellar motility and surfactant production, facilitates collective spreading across surfaces [60]. Twitching, mediated by the extension, attachment, and retraction of type IV pili, supports short-range movement on solid substrates [61].

The physical environment further constrains bacterial growth through surface topology, hydrodynamic forces, and matrix-mediated coupling [62]. For example, surface roughness affects cell adhesion and colony spreading, whereas hydrodynamic forces, such as evaporation-induced flows, critically shape spatial organization. Phenomena such as the coffee-ring effect and Marangoni convection influence initial cell deposition during surface-associated growth, thereby affecting intermixing, plasmid transfer, and the spread of antibiotic resistance [63]. Together, mechanical stresses, active motility, and environmental constraints generate spatial heterogeneity within communities by creating localized variation in nutrient availability, mechanical pressure, and signaling environments. This heterogeneity fosters diverse physiological cell states, where subpopulations exhibit distinct growth rates, metabolic activities, and stress responses, thereby allowing for adaptive community dynamics [64].

### Social interactions

Bacterial communities are shaped by complex networks of social interactions that allow cells to influence and respond to their neighbors. These social interactions can be broadly categorized as competition, cooperation, and communication [71]. Competition refers to actions in which one individual inhibits another from gaining an advantage [72]. Bacteria employ three main types of weapons in competition—chemical, mechanical, and biological—each operating across different spatial ranges [39]. In chemical warfare, bacteria secrete diffusible toxins that act over long distances to inhibit rivals. For example, *P. aeruginosa* secretes pyocyanin, a blue pigment toxin that damages the cellular components of *Staphylococcus aureus* and other competing species [73]. Mechanical weapons, by contrast, require direct physical contact. A notable example is the type VI secretion system (T6SS), a nanomachine used by Gram-negative bacteria to inject toxins into neighboring cells upon contact [74]. Bacteria further expand their competitive arsenal through biological weapons, particularly bacteriophages (viruses that infect bacteria). By strategically deploying phages, bacteria carrying these dormant viral elements can selectively eliminate competing strains while maintaining immunity through strain-specific resistance mechanisms [75–77]. In addition to direct inhibition, bacteria also compete through resource and space exploitation, outgrowing others by rapidly consuming available nutrients or efficiently occupying favorable spatial niches [5]. The integration of chemical, mechanical, and biological weapons, as well as resource and space exploitation, enables bacteria to implement sophisticated competitive strategies across spatial and ecological scales.

Cooperation refers to behaviors in which one individual benefits others. It is commonly seen in producing public goods, which are metabolites, enzymes, or compounds released into the extracellular environment for communal use [42, 78]. For example, *B. subtilis* has been found to segregate into three distinct subpopulations—matrix non-producers, EPS-producers, and generalists—to perform complementary roles in biofilm formation, a phenomenon known as division of labor [79]. Cross-feeding is another form of cooperation, where one species consumes metabolites produced by another, leading to unidirectional or bidirectional dependency that enhances survival [42, 44]. The spatial organization within microbial communities facilitates cooperation by preventing the diffusion of public goods away from interaction partners, thereby increasing their local concentration [2, 42]. For example, in *P. aeruginosa* biofilms, iron chelators (i.e. public goods) are preserved through specific spatial arrangements that ensure their accessibility to neighboring cells [80]. Similarly, spatial segregation—whether in radially expanding colonies or mature biofilms—has been shown to promote antibiotic resistance by creating protective zones where cells collectively produce and share public goods (e.g. EPS, resistant enzymes) [53, 81]. However, cooperative systems are vulnerable to exploitation by cheaters (or “free-riders”)—individuals that benefit from public goods without contributing to their production [82]. Such exploitation can lead to the collapse of cooperative behaviors and a reduction in the population’s overall fitness. In addition to cheaters, environmental fluctuations also challenge the stability of cooperative behaviors. Micali *et al*. showed that cooperative networks among species may collapse during ecological fluctuations, as only a small fraction of the bacterial population resumes growth through cross-feeding after a nutrient shock [83]. This collapse occurs because cross-feeding is highly sensitive to changes in nutrient availability, and only those bacteria well positioned to receive metabolic by-products can survive. Although spatial structure is typically thought to promote cooperation among bacteria, recent findings have challenged this view. Luo *et al*. found that spatial structure can suppress cooperation during colony range expansion by prolonging swarming and increasing vulnerability to cheater invasion [84].

Communication involves the exchange of information between cells through the production, release, and detection of signaling molecules, allowing bacteria to sense population density and coordinate behaviors. One of the best-known communication mechanisms bacteria employ is quorum sensing (QS), a molecular signaling network based on the production and detection of autoinducers [85]. Once a critical cell density is reached, QS triggers coordinated behaviors such as biofilm formation, virulence factor expression, and group motility [45]. QS operates in conjunction with chemotactic signaling, whereby individual cells navigate chemical gradients to seek favorable conditions or avoid harmful environments [42, 86]. These signaling mechanisms allow bacteria to locate metabolite-producing cells, recognize compatible metabolic partners, or evade competitors by interpreting spatial chemical cues [42, 87]. Recent evidence reveals that some bacteria can directly compare chemical gradients across their cell bodies to guide movement. For example, *P. aeruginosa* employs pili-based mechanisms to compare chemoeffector concentrations between its poles during surface-associated growth [88]. This direct gradient sensing challenges the long-held assumption that bacterial cells are too small to resolve spatial differences in chemical signals.

## IbM in studying bacterial interactions

Individual-based modeling (IbM) is a bottom-up approach initially developed for ecological studies at higher trophic levels. As ecological principles are increasingly applied in microbiology studies and significant advances are made in molecular biology and biochemistry, IbM has become an important tool for uncovering microscopic mechanisms underlying population-level dynamics [36, 89]. Concurrently, various modeling approaches have been developed to characterize bacterial community dynamics across multiple spatial and temporal scales.

### Overview of modeling approaches

**Figure 2:**
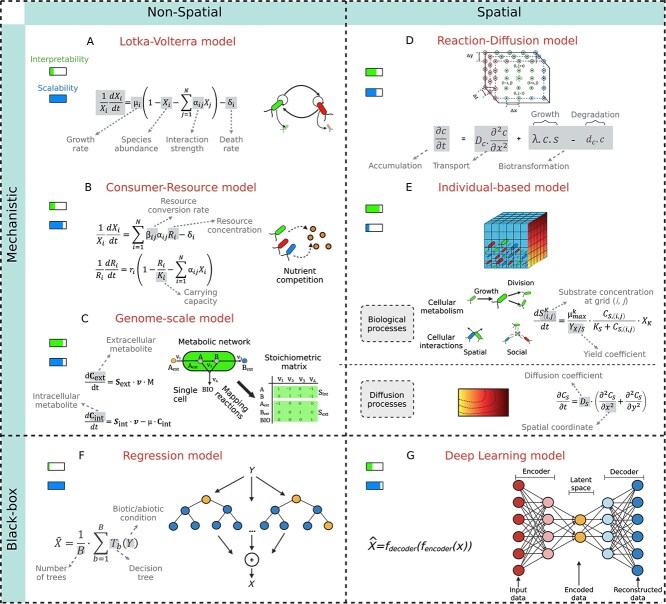
Modeling bacterial interactions across scales. (A–F) Models used to describe microbial interactions are depicted with conceptual diagrams that represent the key processes and interactions across different spatial scales. Mechanistic non-spatial models, ranging from simpler, aggregate descriptions, such as (A) Lotka–Volterra and (B) consumer-resource model, to (C) genome-scale metabolic models (GEM) that reconstruct the metabolic network of a microbe. Finer spatial details can be further incorporated by (D) reaction–diffusion model and (E) individual-based model (IbM). In contrast, black-box models, such as (F) regression models and (G) deep learning models, rely on statistical or computational methods to infer patterns and predict microbial behaviors.

Mathematical modeling provides a structured framework for understanding bacterial interactions across scales. Broadly, modeling approaches in microbiology can be classified as mechanistic or black-box. Mechanistic models use detailed physiological information and the first principles of mathematical reasoning to determine links between intrinsic and extrinsic factors and microbial fitness [90]. Non-spatial mechanistic models, such as the generalized Lotka–Volterra approach [91], have been developed to describe interspecies population dynamics using constant interaction coefficients (Fig. 2A) [92–94]. MacArthur’s consumer-resource model extends this concept by including nutrient dynamics [95]. Top-down extensions to these models further address the stochasticity of intrinsic and extrinsic factors by integrating probabilistic components into the framework (Fig. 2B) [96–100]. To better understand the mechanistic basis of microbial interactions, genome-scale metabolic models (GEMs) reconstruct the metabolic network of a microbe using constraint-based methods. These models allow quantitative predictions of growth rates and metabolic fluxes that influence intercellular interactions [101, 102] (Fig. 2C). In contrast, spatial mechanistic models aim to account for environmental heterogeneity, which plays a critical role in shaping bacterial communities. For example, reaction–diffusion models use partial differential equations (PDEs) to describe biomass movement and nutrient transport in heterogeneous environments (Fig. 2D) [103]. However, these models often neglect individual-level variability and the diverse adaptive strategies employed by bacterial cells in response to changing environmental conditions. IbM addresses these limitations by explicitly modeling the traits and behaviors of individual cells. This bottom-up approach allows population-level properties to emerge from localized cell–cell and cell–environment interactions (Fig. 2E). Nowadays, IbM’s capabilities are further expanded through integration with GEMs. One example is BacArena, a simulation approach that combines flux balance analysis with IbM to model metabolic interactions at the single-cell level [104]. In BacArena, each organism is explicitly represented on a 2D grid, with both its metabolism and movement tracked individually. In contrast, COMETS provides an alternative approach for modeling spatial metabolic interactions. Rather than tracking the metabolic activities of individual cells, COMETS simulates biomass and metabolite dynamics as continuous fields propagating through discretized spatial compartments [105]. These two approaches to integrating cellular metabolism into spatial modeling reflect a trade-off between metabolic resolution and computational efficiency. Additionally, Table 1 provides feature comparisons among IbM toolkits for simulating bacterial communities.

**Table 1:** Feature comparison among IbM toolkits for simulating bacterial communities.

**Features**		**CellModeller**	**iDynoMiCS 2.0**	**NUFEB**	**BacArena**	**Simbiotics**	**Flow-Lenia**	**McComedy**	**BSim 2.0**	**Gro** **2.0**	**Vivarium**	**ACBM**
**Agent**	Chemotaxis		X	X	X	X	X		X	X		X
	Motility	X	X	X	X	X	X		X	X		X
	Differential equation–based dynamics	X	X	X	X	X	X	X	X	X	X	X
	Gene regulatory network	X				X			X	X	X	X
	Membrane transport		X		X	X			X	X		
	Metabolism				X	X				X	X	X
**Environment**	Geometry	2D/3D	2D/3D	3D	2D	2D/3D	2D	2D/3D	2D/3D	2D	2D	3D
	Diffusion	X	X	X	X	X	X	X	X	X	X	X
	Fluid dynamics			X		X	X		X	X		
	EPS		X	X		X						
**General**	Interactive GUI	X				X	X	X				X
	Microscopy image processing					X						
	Programming language	Python	Java	C++	R	Java	Python	Java	Java	C++	Python	Java/MATLAB
**Reference**		[4, 75, 115]	[33, 156–159]	[112, 121, 160]	[102, 161]	[113, 162, 163]	[164]	[165]	[166]	[167, 168]	[169, 170]	[161, 171]

X marks a feature being present.

In contrast to mechanistic models, data-driven approaches (i.e., black-box modeling) predict bacterial dynamics without relying on explicit biological mechanisms. These approaches have gained popularity with the rise of high-throughput sequencing and omics technologies (e.g. 16S rRNA amplicon sequencing and shotgun metagenomics) [10, 106]. Machine learning methods such as random forests [107], multiple kernel learning [108], and neural networks [109] can be applied to microbial community data to infer relationships and patterns. When applied to spatially resolved bacterial community data, advanced machine learning architectures, such as convolutional neural networks and graph neural networks, enable accurate predictions while preserving spatial dependencies [106, 110] (Fig. 2F–G).

### Functionalities and principles of IbM

**Figure 3:**
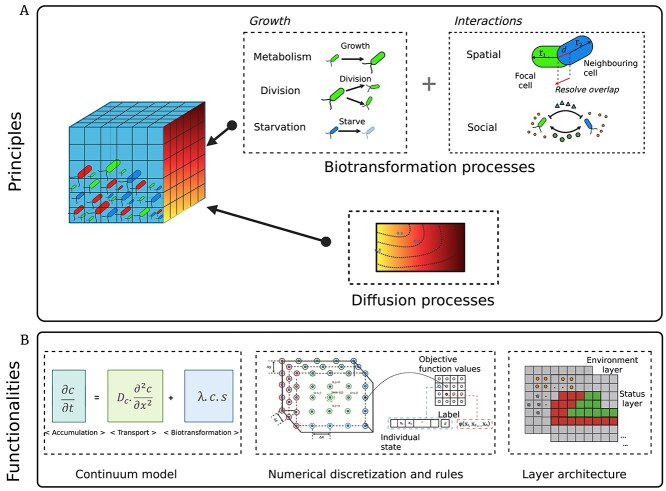
Schematic illustration of IbM in studying bacterial interactions. (A) Individual bacterial cells are represented as entities on a spatial grid, with nutrients and chemical signals diffusing around them. Simulated cells locally uptake available nutrients to grow (cellular metabolism) and engage in interactions with their immediate neighbors (interactions). (B) The underlying principle of bacterial dynamics is that biomass accumulation results from the combined effects of biotransformation (i.e. growth) and transportation (i.e. diffusion). In IbM, discrete and continuous models are integrated numerically to describe bacterial growth and substrate (and/or toxin, signals) diffusion using the “hybrid Eulerian-Lagrangian approach.” Another characteristic of IbM is its layered architecture. The interplay between nutrient (and other chemical) diffusion and bacterial spatial interactions is formulated in IbM by discretizing both spatial and temporal domains.

Mainstream IbM adopts the object-oriented programming principle, whereby each bacterium is modeled as an autonomous object with defined traits and behaviors, which govern its interactions based on internal state and local environmental conditions [111, 112]. The number of objects can range from a few hundred to several million, depending on the complexity of the system being modeled [113]. IbM captures bacterial dynamics through two main processes: bioltransformation and diffusion (Fig. 3A). Biotransformation processes include cellular metabolism (e.g. nutrient uptake, growth, maintenance, division, and starvation) and interactions (i.e. spatial and social) [114]. Diffusion processes describe the movement of extracellular chemical substances such as nutrients, signals, and toxins (Fig. 3A). These processes are integrated in IbM using a hybrid Eulerian–Lagrangian approach, which combines a continuous representation of environmental gradients (Eulerian) with discrete modeling of individual cells (Lagrangian). Typically, this integration employs PDEs to resolve diffusion fields at the environmental scale, while adopting discrete rules and ordinary differential equations to update individual cellular states and positions (Fig. 3B). A key advantage of this hybrid approach is its ability to accurately represent the natural temporal hierarchy of bacterial systems, wherein rapid molecular diffusion (~1 s) and slower cellular processes (e.g. nutrient uptake in *E. coli* occurs over 2~3 min) together drive emergent community behaviors [38, 115].

In IbM, space can be represented either discretely or continuously. In discrete IbM, the simulation space is discretized into fixed-size grid units, and bacterial cells are assigned to specific grid positions [114, 116]. Interactions are calculated within or across neighboring grids, which simplifies computational tasks such as neighbor identification and metabolite exchange. This simplification makes discrete models efficient for large-scale simulations, but introduces limitations such as restricted movement directions and challenges in modeling irregular cell shapes [23, 117]. Continuous IbM, by contrast, allows cells to occupy any position within a continuous spatial domain and move freely [118]. This representation is advantageous in scenarios requiring high spatial resolution, such as heterogeneous or non-uniform environments (e.g. biofilm-forming systems) [119]. However, continuous-space models require more detailed algorithms for collision detection and interaction, thereby increasing computational costs [26].

Spatial interactions in IbM are implemented using either discrete rules or continuum mechanics (Fig. 3B). The discrete rule-based approach applies predefined behavioral rules—e.g. modeling growth-induced pressure release through “cell shoving” rules, in which dividing cells displace adjacent neighbors to avoid spatial overlap [23, 27]. This approach is computationally efficient for modeling threshold-based behaviors (e.g. cellular division upon reaching a critical size or movement in response to local nutrient gradients), but it may oversimplify the physical reality of bacterial interactions [26]. In contrast, the continuum force-based mechanics (FbM) approach treats cells as deformable mass points and calculates net forces based on continuum mechanics equations. Although more computationally intensive, FbM offers higher physical accuracy by capturing elastic deformations, adhesion forces, and complex mechanical interactions within densely packed bacterial communities [6, 35].

IbM models bacterial social interactions through different computational layers. The agent layer represents each bacterium as an object with defined properties such as metabolic state, growth rate, and morphology [111]. The environment layer tracks the spatiotemporal dynamics of environmental factors and cell-secreted molecules, including metabolites, signaling molecules, and toxins. This layered architecture facilitates explicit analysis of bacterial social behaviors under diverse ecological scenarios.

### Applications of IbM in studying spatial interactions

Compared to population-level models, IbM offers two key advantages for studying spatial interactions. First, it provides a natural framework for simulating spatial heterogeneity—a key driver in forming localized micro-niches. Within such niches, populations can specialize in resource utilization and maintain functional diversity. Second, by integrating physical structure with biological function, IbM captures the dynamic feedback between spatial organization and metabolic processes [9]. Spatial organization shapes nutrient uptake and metabolite exchange, while metabolic activity drives localized growth and structural adaptation within the biofilm [2, 9]. Through detailed spatial modeling, IbM reveals mechanisms spanning multiple scales. At the cellular scale, IbM illustrates how variation in cell division, packing density, and self-organization leads to emergent spatial patterns (e.g. fractal colony patterns). At the population scale, IbM elucidates how nutrient gradients, metabolite diffusion, and social behaviors collectively shape community architecture and functional resilience. Through this multiscale integration, IbM provides a mechanistic framework for studying spatial dynamics in bacterial communities. Table 2 provides a non-exhaustive list of IbM applications for studying bacterial interactions.

#### Cellular mechanics

Cell-to-cell mechanical interactions shape the spatial organization of bacterial communities. A key factor governing these interactions is cell morphology, which influences how cells pack, align, and exert forces on one another. Using IbM simulations with experimental validation, Smith *et al*. demonstrated that cell morphology strongly affects biofilm architecture [58]. Their models predicted that shorter, coccoid-shaped cells tend to occupy the upper layers of biofilms, whereas elongated, rod-shaped cells dominate the basal regions and edges. This stratified distribution arises from differences in packing density and mechanical stability, with elongated cells aligning along their length to promote vertical stacking and structural integrity. Further evidence of cell morphology–dependent spatial patterning was provided by Sreepadmanabh *et al*., who showed that the cell aspect ratio (i.e. the ratio of a cell’s length to its width) directly influences colony morphology [57]. Rod-shaped bacteria (i.e. high aspect ratio) tend to produce daughter cells aligned along their length, leading to the formation of elongated colonies. In contrast, spherical cells (i.e. low aspect ratio) divide in random orientations, resulting in more rounded, 3D structures.

**Table 2:** A non-exhaustive list of IbM applications for studying bacterial spatial and social interactions.

**Interactions**			**Entities**	**Purposes**	**Experimental validation techniques**	**Simulation platform**	**References**
**Spatial**	Cellular mechanics	Shape (or aspect ratio)	*E. coli strains with defined MreB mutations altering shape (WT, A53S, A53K)*	To investigate how bacterial cell shape influences spatial organization and fitness in microbial colonies	Confocal and epifluorescence microscopy; automated image analysis	--	[58]
			*E. coli* (high aspect ratio) and *Enterococcus faecalis* (low aspect ratio)	To investigate how cell shape affects bacterial growth in a 3D gut mucosal microenvironment	Inverted laser-scanning confocal microscope	CellModeller	[57]
	Active motility	Swimming	*Acinetobacter baylyi* and *E. coli*	Mechanical interactions between motile and non-motile bacterial species create flower-like patterns in mixed colonies	Time-lapse confocal microscopy	--	[66]
		Swarming	*P. aeruginosa* PA14; wild-type, cheaters (lasR/rsaL mutants), hyperswarmers (fleN mutant)	To investigate how spatial range expansion impacts the stability of cooperative swarming in *P. aeruginosa*	Fluorescent labeling; confocal microscopy; whole-genome sequencing; growth and motility assays; drop collapse assay	--	[84]
			*C. ochracea* (ATCC 27872)	To investigate the physical rules governing the dispersal of T9SS-driven bacterial swarm	Time-lapse stereoscope; AI-based image analysis	--	[120]
		Twitching	--	--	--	--	--
	Environmental forces	Fluid flow	*Vibrio cholerae* biofilms; WT*, ΔrbmA, and inducible strains; extracellular matrix protein RbmA	To uncover how mechanical cell–cell interactions drive 3D order and architecture in growing biofilms	Live confocal fluorescence microscopy; 3D image segmentation and tracking		[56]
			--	To investigate how EPS affects biofilm mechanics and its deformation in flow conditions.	--	NUFEB	[121]
		Surface-associated forces	*E. coli* K12 colonies (strains EQ54, EQ59)	To elucidate how emergent nutrient gradients, mechanical constraints, and metabolism shape the spatial growth and death zones	Confocal and two-photon microscopy; viability assays in batch cultures	--	[122]
**Social**	Competition	Chemical weapons	*E. coli* (engineered strains with contact-dependent or diffusion-based toxin systems)	To determine how spatial scales of microbial interference (short vs. long range) influence community structure and extinction dynamics during range expansion	Time-lapse fluorescence microscopy	CellModeller	[123]
		Mechanical weapons	*A. baylyi*, *Escherichia coli*; synthetic T6SS+ and T6SS− strains; fast- and slow-lysing T6SS effectors (e.g. *Tae*1, Tse2)	To investigate how the evolutionary design of the T6SS affects its efficiency in microbial competition and how cell lysis overcomes limitations caused by corpse barrier effects	Microfluidic and agarose plate competition assays; fluorescent time-lapse microscopy; propidium iodide staining; Hcp secretion assays; genomic analysis across 466 species	CellModeller	[124]
			*P. aeruginosa PAO1* and *E. coli JKE201*	To compare short-range and long-range weapons’ effectiveness in the spatial environment	Fluorescence microscopy	CellModeller	[125]
		Biological weapons	*E. coli* strains TB204 (donor, GFP/CFP) and TB205 (recipient, RFP); conjugative plasmid R388; lytic phage T6	To demonstrate that phage predation increases plasmid-encoded antibiotic resistance transfer by reshaping microbial spatial organization during surface-associated growth	Confocal laser-scanning microscopy; surface-associated growth assays	CellModeller	[77]
	Cooperation	Division of labor	*B. subtilis* (wild-type, Δeps and ΔtasA mutants)	To assess the evolutionary and functional advantages of phenotypic versus genetic division of labor during biofilm matrix production in *B. subtilis*	Confocal microscopy; flow cytometry	--	[79]
			*Pseudomonas stutzeri AN10*	To investigate how substrate concentration and toxicity shape the structure of the microbial communities engaged in metabolic division of labor	Fluorescence microscopy	Gro	[126]
		Cross-feeding	Synthetic *E. coli* communities of two auxotrophic strains (ΔproC and ΔtrpC)	To quantify the spatial scale of microbial interactions and show how short-range metabolite exchange limits cooperative growth and community function	Time-lapse fluorescence microscopy in microfluidic chambers	--	[55]
			*P. stutzeri* A1501	To investigate the effects of spatial patterns on the composition of cross-feeding microbial communities	Confocal laser-scanning microscope	CellModeller	[127]
	Communication	Quorum sensing	--	To investigate a quorum-sensing mechanism at a single-cell level	--	--	[128]
			Synthetic model of QS in *Vibrio fischeri*–like system	To propose and model quorum sensing as a collective environmental sensing mechanism that improves bacterial decision-making by aggregating noisy individual estimates	--	--	[30]
		Chemotactic signaling	*Synechococcus* (picophytoplankton); *Marinobacter adhaerens* HP15 (WT, ΔcheA, ΔfliC mutants)	To determine whether bacterial chemotaxis enhances reciprocal metabolic exchanges with picophytoplankton, challenging the assumption that such interactions are negligible due to small cell size	NanoSIMS for isotope tracking (^13^C, ^15^N); chemotaxis assays (ISCA); flow cytometry; GC–MS metabolomics	--	[86]
		Spatial sensing	Gut microbial strains (generic producers vs. sensitive competitors)	To propose and test the Spatial Sensing (SS) hypothesis, where gut microbes regulate public goods production based on their spatial position relative to the epithelial layer	--	MICRODIMS	[32]

-- marks the detail is not clear.

#### Active motility

Active motility drives the spatial expansion of bacterial communities. IbM has shown how different motility modes generate diverse emergent patterns and influence competitive outcomes in multispecies communities. Swimming motility, driven by flagellar rotation, creates characteristic spatial distributions in mixed populations. For example, Xiong *et al*. used IbM to show that motile *E. coli* cells physically displace non-motile *A. baylyi* cells, leading to the emergence of flower-like patterns in mixed colonies [66]. In contrast to single-cell swimming, swarming involves coordinated multicellular movement. Zdimal *et al*. used IbM, calibrated by time-lapse imaging and particle image velocimetry, to investigate swarming dynamics in *Capnocytophaga ochracea* (an anaerobic oral bacterium) [120]. Their results showed that local cell alignment and density-dependent speed govern phase transitions during swarm development, producing macroscale patterns such as radial bursts, wave-like fronts, and dispersed microcolonies.

For twitching and other surface-associated motility mechanisms, IbM applications have been more limited. Future advances in IbM would benefit from explicitly incorporating these mechanisms, particularly by modeling the biophysical details of pili-driven movement and its influence on cell–cell interactions during community formation.

#### Environmental forces

Environmental forces, including fluid flow and surface interactions, play a critical role in shaping biofilm development and detachment. Hartmann *et al*. developed an IbM to simulate surface-attached biofilms under varying external flow strengths [56]. Their model, which incorporates cell-to-cell forces, steric repulsion, osmotic pressure, and matrix interactions, showed that higher shear rates produce smaller, more compact biofilms with drop-like morphologies, whereas low-shear conditions favor more expansive structures. For computational efficiency, many hydrodynamic IbMs assume simplified exponential growth kinetics for individual cells and neglect the metabolic heterogeneity observed within biofilms. To address this limitation, Kannan *et al*. developed a 3D IbM that integrates cell metabolism with mechanical interactions to simulate bacterial colony expansion on solid agar [122]. Their simulations revealed that mechanical forces drive radial expansion, while vertical growth is constrained by nutrient diffusion. These findings highlight the combined effects of mechanical forces and metabolic constraints in shaping biofilm architecture. Fluid flow also influences biofilm detachment. Li *et al*. developed a parallel simulation approach (NUFEB) to model biofilm development and detachment under flow conditions [113]. Their approach simulates biofilm formation in two stages: first, fluid flow is applied to a pre-grown biofilm composed of heterotrophs and EPS, producing mature structures such as mushroom-like formations, voids, and channels; second, as flow intensity increases, detachment is induced, during which biological activity is halted and cell movement is governed solely by hydrodynamic forces. Building on this framework, Xia *et al*. showed that higher EPS content enhances biofilm stability by increasing stiffness, shear modulus, and viscoelasticity [121].

In addition to fluid flow, surface-associated forces also influence biofilm development. Tecon *et al*. used IbM to reveal that limited water connectivity in heterogeneous soil environments restricts bacterial motility, thereby prolonging cell-to-cell contact and improving interaction frequency [62]. Additional details for the case studies discussed above are presented in Table 2.

### Applications of IbM in studying social interactions

By explicitly modeling individual cell behaviors, IbM provides mechanistic insight into how spatial organization regulates bacterial social interactions. For competition, IbM shows that spatial structure regulates the efficacy of competitive strategies by influencing diffusion barriers for chemical weapons, targeting efficiency for contact-dependent weapons, and propagation dynamics for biological weapons such as bacteriophages [77, 124, 129]. These spatial effects result in the formation of distinct competitive zones and drive patterns of species distribution and resource allocation. For cooperation, IbM reveals a dual role of spatial structure: it promotes cooperation by facilitating metabolite exchange and public goods retention among neighboring cells, yet it can also hinder cooperation when physical separation or environmental heterogeneity restricts resource diffusion and imposes unequal metabolic costs [22, 84, 130]. For communication, IbM demonstrates that spatial organization governs both signal diffusion and information content, determining whether signals convey local density or site-specific environmental cues [30, 32]. These spatially mediated signaling dynamics allow bacteria to coordinate collective behaviors across different scales.

#### Competition

**Figure 4:**
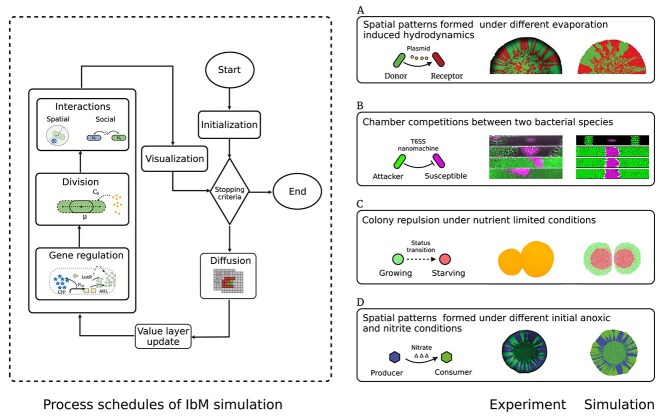
Spatial patterns emerge from IbM simulations. On the left, process schedules are conceptualized from CellModeller [116] and MICRODIMS [23, 29]. Starting with the initialization of cell types and environmental conditions, the simulation progresses through a time-stepped loop that includes nutrient uptake, metabolism, cell growth, division decisions, mechanical interactions, environmental updates, and gene expression regulation. Decision points within the loop determine whether cells divide or the simulation continues to the next time step. (A–D) Spatial patterns emerged from localized individual interactions for different scenarios: (A) metabolic differentiation drives the emergence of the starvation zone in the center of growing colonies (pictures adopted from Tack *et al*. [29] with permission under CC-BY license); (B) droplet evaporation determines the spatial distributions of bacterial cells and subsequently controls the spread of an antibiotic resistance-encoding plasmid during surface-associated growth (pictures adopted from Ruan *et al*. [63] under CC-BY license); (C) chamber competitions between T6SS attacker species and susceptible species lead to distinct growth patterns of dual-species communities (pictures adopted from Smith *et al*. [124] under CC0 license); (D) spatial patterns are formed under different initial anoxic and nitrite conditions by two strains. One strain can reduce nitrate but not nitrite (producer), whereas the other can reduce nitrite but not nitrate (consumer) (pictures adopted from Ciccarese *et al*. [127] under CC-BY license).

Bacteria employ chemical, mechanical, and biological weapons in competition. Chemical weapons, such as antibiotics and bacteriocins, can diffuse over long distances to inhibit rivals [3]. Celik Ozgen *et al*. showed through IbM that diffusible toxins establish spatial interference scales during colony expansion, with higher toxin diffusion rates accelerating the elimination of toxin-sensitive cells [123]. Mechanical weapons, in contrast, require direct physical contact [39]. For example, the T6SS drives a toxin-laden needle into neighboring cells upon contact. However, the efficiency of this contact-dependent inhibition (CDI) is limited by the accumulation of lysed cells (“corpse barriers”) that block further attacks. Combining IbM with microfluidic assays, Smith *et al*. showed that lytic effectors (e.g. *Tae*1) enhance T6SS killing by rapidly lysing target cells, thereby preventing “corpse barriers” formation. Comparative genomic analyses of T6SS-equipped bacteria further reveal that genes encoding these potent lytic (i.e. cell wall–degrading) effectors are widely conserved [124] (Fig. 4B). Building on this, Booth *et al*. investigated how bacterial motility improves the efficacy of CDI mechanisms. Their simulations demonstrated that motility enables attacker cells to move through prey clusters, breaking up static “corpse barriers” and continually forming new attacker–prey contacts. This continual renewal of the contact zone allows rapid retargeting of live cells, amplifying CDI killing efficacy [125].

Spatial constraints introduce additional complexity to bacterial competition. Liu *et al*. investigated how spatial positioning affects competition outcomes between microbial populations [131]. Using IbM, they demonstrated that dispersed spatial colonization improves competitive success. Their findings revealed that populations with higher “space accessibility” can compensate for disadvantages in growth rate or initial abundance, offering insights into how spatial competition drives evolutionary dynamics and maintains biodiversity in structured environments. To quantify this concept, Eigentler *et al*. introduced the “access to free space” (AFS) score, a geometric metric that measures the fraction of the inoculum’s boundary adjacent to a strain’s founder cells. Their findings confirmed that strains with higher AFS scores (i.e. larger boundary shares) expand more rapidly into fresh agar. Conversely, interior cells (i.e. with smaller boundary shares and lower AFS scores) are surrounded by neighbors and cannot expand outward [132]. Similarly, Copeland *et al*. investigated how spatial constraints affect competition among mutual-killing bacterial strains. They found that smaller environments favor strains with slower killing rates, whereas larger environments promote the dominance of faster-killing strains [65]. Bacteria also deploy biological weapons, such as bacteriophages, in competition. Ruan *et al*. studied phage-mediated competition between two *E. coli* strains [77]. Their results showed that phage predation induces spatial reorganization within colonies, shifting zones of rapid proliferation from the periphery to the interior as cells evolve resistance. This spatial adaptation inadvertently increases cell–cell contact, which enhances plasmid transfer and accelerates the spread of antibiotic resistance. These findings highlight how phage predation mediates bacterial competition by imposing selective pressures and reshaping spatial reorganization. In addition to direct inhibitory mechanisms, resource competition is an important driver for bacterial spatial dynamics. Mitri *et al*. employed an IbM to assess how nutrient availability influences competition in *P. aeruginosa* colonies. Their simulations revealed that nutrient limitation induces spatial segregation of competing strains (i.e. each genotype clustering into its own clonal patch), which in turn reduces intermixing and leads to competitive exclusion [129]. Tack *et al*. expanded on this by incorporating a reduced stoichiometric network of *E. coli* metabolism into an IbM. Their study demonstrated that local variations in nutrient and oxygen availability lead to stratification in submerged *E. coli* colonies [133] (Fig. 4C). Ma *et al*. further showed that nutrient competition between *Pseudomonas* strains results in spatial segregation within microchannels, significantly reducing plasmid transfer rates and thereby limiting horizontal gene transfer. This reduction in genetic exchange constrains the adaptive potential of bacterial populations, thus restricting their ability to acquire beneficial traits (e.g. antibiotic resistance) [134].

#### Cooperation

Bacteria rely on cooperative interactions to improve collective survival and resource utilization. Dragoš *et al*. used IbM to investigate the division of labor during *B. subtilis* biofilm formation [79]. In their model, each cell is represented as a disc on a 2D toroidal surface that grows, divides, and secretes EPS components. Their model characterized different subpopulations by assigning distinct functional roles: some cells specialized in producing EPS, others in producing *TasA* (a protein essential for biofilm structure), while a third group consisted of non-producers. Their simulations revealed that an imbalanced strain ratio naturally emerges due to unequal benefits conferred by EPS and *TasA*. This genetic division of labor alleviates metabolic burdens and enables more efficient biofilm formation. Wang *et al*. investigated how communities engaged in metabolic division of labor respond to variations in substrate concentration and toxicity [126]. Using IbM and synthetic consortia experiments, they revealed that higher substrate availability and toxicity favor the population performing the first metabolic step (the “Detoxifier”) by increasing metabolite flux and reducing toxic effects. Their findings highlight that environmental stresses can reshape the metabolic division of labor, driving changes in community composition and spatial structure within microbial habitats. Furthermore, Dal Co *et al*. studied cross-feeding interactions of *E. coli* communities [55]. Their model simulated the exchange of amino acids (a key communal metabolite) between individual cells, showing that high uptake rates and dense cell packing are critical for determining the range and efficiency of cooperative interactions. Their findings emphasize the role of physical proximity and uptake efficiency in maintaining cooperation in spatially structured environments. Ciccarese *et al*. extended this investigation by studying the effects of temporal environmental fluctuations (e.g. redox conditions) on cross-feeding [127]. Their IbM showed that changes in nitrate and nitrite availability significantly altered the spatial organization and stability of cooperation in surface-associated bacterial colonies, producing irreversible shifts in community composition and metabolic function (Fig. 4D). In addition to environmental fluctuations, social cheating also challenges the stability of cooperation. Luo *et al*. used IbM to study cooperation during colony range expansion and found that spatial structure can suppress cooperation by prolonging cooperative swarming, thereby increasing vulnerability to cheater invasion [84]. In parallel, to generalize the impact of molecular dynamics on community-level properties, Vliet *et al*. developed an IbM that combined a biophysical model for deriving local interaction rules with a graph-based approach for predicting community properties [22]. Applied to mutualistic cross-feeding communities, their model further revealed that spatial structure may hinder community function by constraining cooperative interactions. Cooperation is important not only in metabolic exchanges but also in bacterial defense mechanisms. Granato *et al*. used IbM to show how EPS-producing bacteria protect their community against T6SS attacks [135]. Their model demonstrated that bacteria not only share protective EPS molecules but also form defensive barriers to shield vulnerable neighbors—a cooperative defense mechanism known as “flank protection.”

#### Communication

Bacterial communication involves intricate molecular signaling systems that enable cells to coordinate collective behaviors. Melke *et al*. developed an IbM to study quorum sensing (QS) in heterogeneous bacterial colonies. By explicitly modeling autoinducer diffusion, their simulations showed that spatial organization and colony heterogeneity significantly impact QS activation thresholds [128]. Similar to cooperative systems, QS is vulnerable to cheaters. Hashem and Van Impe built an IbM to investigate the vulnerability of the QS system to such cheating behaviors. They introduced the concept of “the least expensive reliable signal” to infer the optimal metabolic investment by a focal species to resist “cheating” [136]. A key assumption in their model is that cells could accurately estimate population density through local autoinducer concentrations. However, this assumption may not hold in fluctuating environments where signal diffusion coefficients and degradation rates vary. To address this, Moreno-Gámez *et al*. developed an IbM incorporating a gene regulatory network with positive feedback and bistable expression states [30]. By treating gene product exchange as an evolvable trait, they showed that communication can evolve into a collective sensing strategy that allows bacteria to aggregate individual estimates and improve community responses. In addition to QS, Raina *et al*. used IbM to investigate how chemotaxis functions as a sensing mechanism regulating bacterial-phytoplankton interactions [86]. Their model and experiments showed that phytoplankton release dissolved organic matter, thereby generating chemical gradients that guide chemotactic bacteria toward nutrient-rich microenvironments. This activity enhances metabolic exchanges and shapes community structure and nutrient cycling in marine ecosystems. Adding further depth, Hashem *et al*. proposed the spatial sensing hypothesis as a complementary strategy to QS and chemotaxis using an IbM framework [32]. By explicitly modeling host-secreted gradients and bacterial public goods regulation, their simulations showed that bacteria could evolve spatially dependent regulation mechanisms to optimize public goods production based on their location within the gut environment. Additional details for the case studies discussed above are presented in Table 2.

Taken together, these studies show how IbM bridges microscale cellular interactions and macroscale community dynamics to uncover regulatory mechanisms that population-level approaches cannot capture. For spatial interactions, IbM reveals how cellular mechanics, motility, and environmental forces collectively shape community architecture. For social interactions, IbM uncovers how spatial structure regulates competitive outcomes, stabilizes cooperation, and structures communication networks. Despite these advances, current IbM implementations remain limited by simplified bacterial physiology, static environmental conditions, and high computational demands [37, 137]. Future improvements can focus on integrating detailed physiological models with environmental physics [102, 138], developing multiscale modeling frameworks [114, 139], and incorporating evolutionary dynamics to transform IbM from a descriptive to a reliable predictive tool for engineering microbial communities across diverse applications [140].

## Calibrating IbM with experimental data

Integrating IbM with experimental data (and/or observations) is essential for developing reliable simulations of bacterial community dynamics. Modern high-resolution experimental techniques (50–200 nm spatial resolution) provide the data needed to constrain and validate these models [12, 13]. Confocal and fluorescence microscopy enable precise measurements of single-cell properties, including cell size [55], shape [57, 58], and intracellular content [134]. When combined with time-lapse imaging, these techniques reveal dynamic processes such as growth patterns, cell division rates, and colony expansion [55]. Genetic tracking using fluorescent markers provides additional insight by allowing researchers to monitor specific populations within mixed communities, thereby validating model-predicted lineage structures against empirical observations.

Flow cytometry provides complementary high-throughput measurements of bacterial size distributions and population dynamics. For example, García *et al*. used cytometry data to parameterize an IbM for *Pediococcus acidilactici* by estimating growth rates, size variability, and division thresholds [141]. The calibrated model successfully reproduced the experimentally observed volume distribution. Microfluidic platforms further enhance experimental integration by allowing precise manipulation of environmental conditions (e.g. nutrient gradients and shear forces) under real-time observation [142–144]. For example, microfluidic “mother machines” allow long-term tracking of individual bacterial lineages [145], whereas flow cells enable studies of biofilm formation under controlled shear stresses [146]. The quantitative data collected in these environments, such as single-cell geometry (e.g. length, width), division and growth rates, nutrient and signal concentration gradients, and molecular diffusion and degradation constants, obtained in these controlled environments are particularly valuable for calibrating IbM predictions about the effects of physical and chemical factors on community development. Biochemical assays further improve model parameterization by quantifying molecular interactions that drive community behaviors. By measuring the production and effects of signaling molecules, toxins, and other chemical compounds, these assays provide essential parameters for modeling chemically mediated interactions between bacteria [4, 135].

Despite these advances, accurate parameter estimation remains challenging. For example, high-resolution imaging often alters physiological conditions, as the intense illumination required for super-resolution fluorescence can induce phototoxic stress that slows cell division and changes physiology [147]. Similarly, confining cells within microfluidic assays may produce growth dynamics that differ from those of natural biofilms [148]. Moreover, chemical-flux estimates obtained from different analytical platforms (e.g. Liquid Chromatography-Mass Spectrometry, Gaschromatography-mass spectrometry, or mass spectrometry imaging) often differ by orders of magnitude, which introduces great uncertainty into modeled uptake and secretion rates [149]. Therefore, integrating high-resolution physical and chemical data with rigorous uncertainty quantification is essential to calibrate IbMs.

## Concluding remarks and future perspectives

IbM serves as a powerful hypothesis-generating tool for uncovering the fundamental principles of microbial interactions. For spatial interactions, IbM reveals how bacterial growth kinetics, mechanical force, spatial constraints, and environmental factors collectively shape biofilm architecture, niche partitioning, and emergent structural organization. For social interactions, IbM provides mechanistic insights into bacterial behaviors, elucidating how competition, cooperation, and communication are regulated within spatially structured environments. By operating at the single-cell level, IbM enables the integration of bottom-up approaches with top-down community-level observations. These simulated experiments, though sometimes imperfect, advance our understanding of complex bacterial communities by uncovering mechanisms that are difficult to isolate experimentally. Furthermore, the insights gained from IbM provide a foundation for practical applications in microbial consortia engineering [136]. These simulations support efforts to optimize spatial arrangements for efficient cross-feeding [123], design interaction networks that remain robust under environmental perturbations [31], and develop control strategies to maintain desired community compositions in applied settings [150, 151].

Nevertheless, limitations persist that constrain the broader applicability of IbM. First, scaling IbM to represent large, taxonomically and functionally diverse microbial communities remains computationally challenging, particularly when detailed molecular mechanisms are retained. Potential solutions include developing efficient numerical algorithms [23] and adopting dimension-reduction approaches that lower the computational burden inherent to IbMs [31, 139]. Second, integrating multiscale experimental data into IbM presents both a challenge and an opportunity. Recent experimental advances—particularly in single-cell transcriptomics, metabolomics, and spatially resolved imaging—provide unprecedented resolution of cellular heterogeneity and microenvironmental conditions. However, translating these high-dimensional datasets into useful model parameters requires robust models for parameter estimation and cross-validation against independent experimental measurements [102]. Third, the stochastic nature of microbial interactions and experimental uncertainties requires rigorous validation and uncertainty quantification to ensure model reliability [36]. Bayesian parameter estimation using Markov Chain Monte Carlo methods provides posterior distributions for probabilistic inference [152]. Sensitivity analysis, including local (e.g. one-at-a-time) and global (e.g. Sobol indices, Morris screening) approaches, identifies key parameters influencing model outcomes [153]. Furthermore, uncertainty propagation methods such as linear approximation, sigma point methods, and polynomial chaos expansion offer trade-offs in computational cost and robustness [154]. Fourth, environmental heterogeneity remains difficult to represent in current IbMs. Future developments should explicitly incorporate environmental fluctuations and spatial heterogeneity to better reflect natural ecosystems. This includes adding spatiotemporally explicit resource landscapes, physical structures, and microenvironmental gradients that characterize natural microbial habitats [155].

## Acknowledgements

We thank Prof. Bas Teusink for hosting the research stay during the revision. We thank Dr Pranas Grigaitis, Francesco Moro, Zixu Wang, Luis Salinas, and Sabine Michielsen (*SysBioLab*, Vrije Universiteit Amsterdam) for their valuable comments and discussion. We also thank Dr Chen Long, Hunan University of Technology, for his helpful advice on improving the figure layouts. We further thank Ms. Kaiyu Zhang for proofreading the manuscript. All figures were created with BioRender (https://www.biorender.com/).

## Data Availability

No datasets were generated in this work.
